# Towards the development of continuous, organocatalytic, and stereoselective reactions in deep eutectic solvents

**DOI:** 10.3762/bjoc.12.258

**Published:** 2016-12-05

**Authors:** Davide Brenna, Elisabetta Massolo, Alessandra Puglisi, Sergio Rossi, Giuseppe Celentano, Maurizio Benaglia, Vito Capriati

**Affiliations:** 1 Dipartimento di Chimica, Università degli Studi di Milano, Via C. Golgi 19, I-20133 Milano, Italy; 2 Dipartimento di Scienze Farmaceutiche, Università degli Studi di Milano, Via Mangiagalli 25, 20133 Milano, Italy; 3 Dipartimento di Farmacia–Scienze del Farmaco, Università di Bari “Aldo Moro”, Consorzio C.I.N.M.P.I.S., Via E. Orabona 4, I-70125 Bari, Italy

**Keywords:** continuous process, DES, organocatalysis, proline, stereoselective aldol reaction

## Abstract

Different deep eutectic solvent (DES) mixtures were studied as reaction media for the continuous synthesis of enantiomerically enriched products by testing different experimental set-ups. L-Proline-catalysed cross-aldol reactions were efficiently performed in continuo, with high yield (99%), *anti-*stereoselectivity, and enantioselectivity (up to 97% ee). Moreover, using two different DES mixtures, the diastereoselectivity of the process could be tuned, thereby leading to the formation, under different experimental conditions, to both the *syn*- and the *anti-*isomer with very high enantioselectivity. The excess of cyclohexanone was recovered and reused, and the reaction could be run and the product isolated without the use of any organic solvent by a proper choice of DES components. The dramatic influence of the reaction media on the reaction rate and stereoselectivity of the process suggests that the intimate architecture of DESs deeply influences the reactivity of different species involved in the catalytic cycle.

## Introduction

The aldol reaction is a powerful synthetic tool to create new C–C bonds [1]. It offers several possibilities to control the stereochemical outcome of the process and to afford stereochemically defined chiral products [2]. Among all the possible options, the L-proline-catalysed stereoselective cross-aldol reaction remains the greener choice. After the pioneering works by List and Barbas [3], a huge effort was made by the scientific community to improve both the yield and the stereoselectivity of the reaction. The most explored strategies involve the development of a new class of catalysts (mainly prolinamide derivatives) [4–6], the study of additives in combination with proline itself [7–13], and the use of unusual reaction media [14–19].

In this context, it was recently reported that L-proline-catalysed direct aldol reactions may be successfully carried out also in deep eutectic solvents (DESs) [20–22]. Recently, our group reported on the possibility of running organocatalyzed, stereoselective reactions in DESs, promoted by an enantiopure primary amine, with advantages in terms of reaction sustainability. In particular, the possibility to strongly reduce the amounts of organic solvent and the recyclability of the catalyst were demonstrated [23]. Moreover, in this approach, no structural modification of the precious chiral catalyst was necessary.

A well-explored strategy aimed at positively realizing the recovery and the reuse of the catalyst is represented by the immobilization of the catalytic species [24–27]. Synthetic modifications of the original catalyst, however, are required in order to attach the catalyst to the material of choice. The aim of the present study was to develop a catalytic system working in continuo, whereas DES acts at the same time as catalyst trap and as reaction medium, immiscible with the organic reactants. The main advantage of this approach is that the catalyst (i.e., L-proline) would be kept in an environmentally benign reaction medium, without the need of any synthetic modification. Of note, in the herein proposed system, readily assembled using standard glassware, the use of the organic solvent, both for the reaction and for the isolation process, would be strongly reduced or even, ideally, eliminated.

## Results and Discussion

Among the plethora of possible DES mixtures [28–33], based on our previous experience [34–39] and preliminary studies on the physicochemical properties of DES combinations, we decided to focus our attention on the use of a few choline chloride (ChCl)-based eutectic mixtures as reaction media (Table 1) [40].

**Table 1:** ChCl-based eutectic mixtures used in the present work.

DES	Components	Molar ratio

DES A	ChCl/urea	1:2
DES B	ChCl/urea/H_2_O	1:2:1.5
DES C	ChCl/urea/H_2_O	1:2:4
DES D	ChCl/fructose/H_2_O	1:1:1
DES E	ChCl/glycerol	1:2

**Scheme 1:**
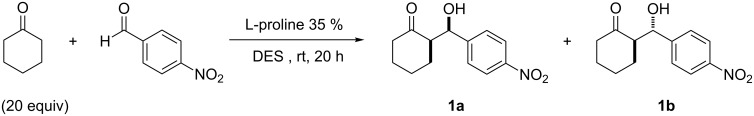
L-Proline-promoted stereoselective aldol reaction in DES.

The behaviour of DES mixtures A–E in the proline-catalysed model aldol reaction between cyclohexanone and 4-nitrobenzaldehyde was preliminarily investigated under standard batch conditions (Scheme 1).

In our hands, the reaction proceeded completely in 20 hours and with high conversion (≥95%) in all tested DESs (A–E, Table 2, entries 1–5). While low diastereoselectivity was observed in DES A (Table 2, entry 1), *anti-*stereoselectivity (up to 85:15) and high enantiomeric excess in favour of the *anti* isomer (up to 92% ee) were instead detected running the reaction in DESs B–E (Table 2, entries 2–5).

**Table 2:** DES screening for the proline-catalyzed in batch aldol reaction.

Entry	DES	Conv. (%)^a^	dr (*anti*:*syn*)^a^	ee % (*anti*/*syn*)^b^

1	A	99	57:43	81/80
2	B	98	82:18	89/69
3	C	96	85:15	92/54
4	D	95	75:25	84/67
5	E	96	70:30	82/67

^a^Conversion and dr were evaluated by NMR technique on the crude reaction mixture; ^b^ee was evaluated by using an HPLC with a chiral stationary phase.

Based on these results, we turned our attention to design and realize a home-made system, to be easily assembled with common glassware, for the continuous synthesis of the aldol product, using a DES mixture as reaction media able to hold back the proline.

**Figure 1:**
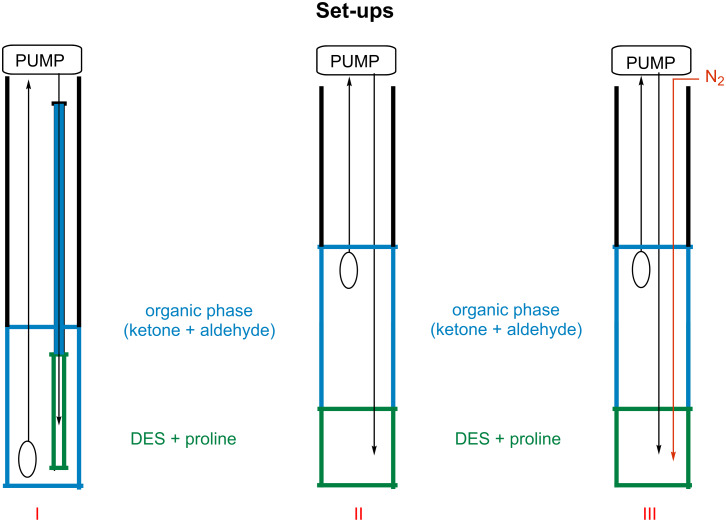
Experimental set-up I: test tube (*d* = 0.5 cm); flow 1 mL/min; DES (1.5 mL); L-proline/DES = 130 mg/mL. Experimental set-up II: test tube (*d* = 2.5 cm); flow 1 mL/min; DES (1.5 mL); L-proline/DES = 130 mg/mL. Experimental set-up III: test tube (*d* = 2.5 cm); flow 1 mL/min; DES (1.5 mL); L-proline/DES = 130 mg/mL.

In these very explorative studies, different experimental set-ups were investigated, focusing especially on some points, such as (a) the phase contact between the organic phase, composed by cyclohexanone and the aldehyde, and the DES phase, (b) the ratio between DES and L-proline, and, finally, (c) the possible interaction between the aldol product and the DES network (Figure 1). Due to its favourable physical and mechanical properties, DES A was selected for the initial screening of the different experimental conditions in continuo.

The first experimental set-up that was studied (Figure 1, I) was built using a test tube of reduced diameter (green color in the picture) containing the DES and L-proline, surrounded by an external, larger cylinder filled with a solution of cyclohexanone and 4-nitrobenzaldehyde. The organic solution, fluxed by a HPLC pump onto the bottom of the internal smaller tube, went back through DES due to the difference in the viscosity of the two phases, thereby generating a upper organic phase (blue in the picture) which finally ended into the organic phase of the larger tube, that was continuously pumped into the DES phase to realize a closed cycle.

In set-up II, the mixture of DES and L-proline was covered with the solution of ketone and aldehyde in a 10 mL graduated cylinder. The organic phase was continuously pumped on the bottom of the DES phase and recirculated (Figure 1, II). In order to improve the contact surface between the two phases and favour the phases interaction, nitrogen was used as a diffusor, thus realizing in set-up III a better mixing of the two phases (Figure 1, III).

By monitoring the transformations performed with the above-described different set-ups, it was observed that both the diastereoselection and the enantioselectivity were constant during the reaction time (Table 3). With set up I (Table 3, entries 1–5), after 20 h, a 39% conversion was reached, while full conversion was obtained after 48 h of reaction. Remarkably, high ee values for the *syn* adduct were observed (up to 94% ee), unfortunately, with a low diastereoisomeric ratio (dr). Using set-up II (Table 3, entries 6 and 7), after 24 h, the conversion was still very low (35%) and the ee for the *syn* aldol was up to 90%, the complete conversion was achieved after 48 h. Interestingly, the analysis of the mass of the crude mixture showed that a part of the product was trapped into the DES phase. In order to quantitatively collect the aldol adduct, the DES was diluted with 1 mL of water and extracted five times with 2 mL of ethyl acetate. Using this procedure, all the aldol adduct was completely recovered.

In the set-up III (Table 3, entries 8–11) the presence of a more efficient phase mixing led to a faster conversion. After only 5 h (Table 3, entry 8), 26% conversion was observed, with interesting diastereoselection and high enantioselection (up to 92% for the *syn* adduct). After 48 h, the aldehyde was almost quantitatively converted into the desired aldol product, with high enantioselectivity for both the *syn (*up to 92%) and the *anti* (up to 90%) isomers.

**Table 3:** Three different set-ups for the aldol reaction in continuo*.*

Entry	Set-up	Time (h)	Conv. (%)^a^	*anti*:*syn^a^*	ee% (*anti*/*syn*)^b^

1	I	20	39	59:41	70/94
2	I	24	47	58:42	68/92
3	I	40	87	55:45	79/92
4	I	48	99	53:47	76/88
5	I	wash^c^	99	52:48	70/84
6	II	24	35	49:51	78/90
7	II	48	96	64:36	84/83
8	III	5	26	62:38	86/92
9	III	24	48	63:37	90/91
10	III	48	90	64:36	84/85
11	III	wash^c^	91	67:33	84/85

^a^Conversion and dr were evaluated after removing cyclohexanone from samples taken at indicated reaction times; ^b^ee was evaluated by HPLC on chiral stationary phase. ^c^in order to wash the pump 2 mL of cyclohexanone were used.

**Scheme 2:**
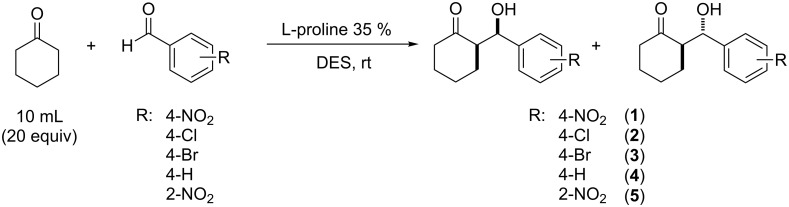
Aldol reaction under continuous flow conditions in DESs.

Having identified the system III as the best experimental set-up, the general scope was briefly investigated by running the reaction with a few different aldehydes and comparing the activities of DES mixtures A and B in the reactions performed in continuo (Scheme 2).

In the case of 4-nitrobenzaldeyde, the use of DES B (a ternary mixture of ChCl, urea and water, 1:2:1.5 ratio) led to impressive results, both in reaction rate and stereoselectivity, compared to the reaction run in DES A (Table 4, entries 1–4). The reaction proceeded completely in only 15 h, and afforded a clean product (aldol **1**, Scheme 2) that was easily isolated by evaporation of excess cyclohexanone, with high *anti-*diastereoselectivity (up to 90:10), and enantioselectivity (up to 92%) for the major *anti* isomer.

**Table 4:** In continuo aldol reactions of different aldehydes in DES A and DES B.

Entry	DES	Aldol	R	Time (h)	Conv. (%)^a^	*anti:syn*^a^	ee % (*anti/syn*)^b^

1	A	**1**	4-NO_2_	5	26	62:38	86/92
2	A	**1**	4-NO_2_	20	48	63:37	90/91
3	B	**1**	4-NO_2_	5	73	85:15	92/70
4	B	**1**	4-NO_2_	15	99	90:10	90/70
5	A	**2**	4-Cl	3	13	71:29	73/78
6^c^	A	**2**	4-Cl	24	99	57:43	73/78
7	B	**2**	4-Cl	3	50	88:12	88/73
8	B	**2**	4-Cl	24	91	80:20	88/77
9	A	**3**	4-Br	24	67	65:35	81/70
10	A	**3**	4-Br	42	99	65:35	80/70
11	B	**3**	4-Br	3	10	70:30	91/85
12	B	**3**	4-Br	24	75	70:30	93/86
13	B	**4**	H	42	20	90:10	87/64
14	B	**5**	2-NO_2_	3	9	90:10	95/50
15	B	**5**	2-NO_2_	24	51	93:7	97/52

^a^Conversion and dr were evaluated after removing cyclohexanone from samples taken at indicated reaction times; ^b^ee was evaluated using an HPLC with a chiral stationary phase; ^c^in this case, it was necessary to use 10 mL of EtOAc to quantitatively recover the aldol adduct (Supporting Information File 1).

By performing the reaction with 4-chlorobenzaldehyde in DES A (entries 5 and 6, Table 4), the desired aldol product **2** was obtained in 99% yield after only 24 h, with up to 73% enantioselectivity for the *anti* isomer. Notably, using DES B (Table 4, entries 7 and 8) a high *anti* diastereoselectivity (up to 88:12) jointly with a very high ee for the major isomer (up to 88% ee) was detected. It is worth mentioning that when working in DES A, the aldol adduct **2** was partially retained in the DES phase and an extraction with ethyl acetate was necessary to quantitatively recover the product. However, as for the reaction in DES B, the whole aldol product was recovered simply by evaporating the organic phase (distilling off the excess of cyclohexanone; for experimental details see Supporting Information File 1).

Analogous results were obtained in the reaction with 4-bromobenzaldehyde. In DES B, the aldol product **3** was isolated in higher yield and stereoselectivity than in DES A (Table 4, entries 9–12; 93% ee for the major *anti* isomer). While the reaction with benzaldehyde led to poor results, the conversion of 2-nitrobenzaldehyde in the expected aldol adduct **5** proceeded in moderate yield (51% after 24 h), but with a remarkable *anti*-diastereoselectivity (93:7) and enantioselectivity (up to 97%).

The different stereoselectivities of the reaction observed in different DES phases could be related to the creation of different tridimensional networks between DES and L-proline, and thus of different chiral reaction environments possibly affecting the stereochemistry of the intermediate species involved in the catalytic cycle [41]. The equilibrating nature of the aldol reaction and the influence of such reversibility on its stereochemical outcome has recently been studied [42]. It has also been reported that the use of additives may have a dramatic influence on the diastereoselectivity and the enantioselectivity in proline-catalyzed aldol transformations [43].

Typically, reactions run in DES mixtures lead to a very clean crude mixture. The recovery of the final aldol adduct can be, indeed, achieved using a reduced quantity of cyclohexanone (12 mL for 1.3 grams of crude aldol), that could be recovered by distillation and reused in new reactions (for experimental details on the product recovery, mass balance and ^1^H NMR spectra of the crude mixture see Supporting Information File 1).

Finally, we also performed preliminary recycling experiments using two different DESs and set-up III. DES mixtures A or B (1.5 mL), containing L-proline (0.35 equiv, 195 mg), previously used for 48 h in the aldol reaction of cyclohexanone with 4-nitrobenzaldehyde, were recycled in the same transformation. At the end of the reaction, the pump was washed with 3 mL of cyclohexanone, in order to recover the product present in the pump system, then the supernatant (cyclohexanone and aldol product) was separated from the DES phase, containing the catalyst, and analyzed. To the DES phase, new reagents (cyclohexanone and aldehyde) were added and the reaction was started again. While the catalytic system in DES A showed a lower activity, thus affording the product in a significant lower yield, the L-proline/DES B system afforded results comparable to the first run, both in terms of chemical yield and stereoselectivity (93% yield, 92% ee for the major *anti* isomer; see Table S2 in Supporting Information File 1).

## Conclusion

In conclusion, the possibility of a continuous, organocatalyzed, stereoselective process in DES was, for the first time, studied and successfully developed. Using different experimental set-ups, it was possible to realize efficient proline-catalysed cross-aldol reactions in continuo with high yield (99%), *anti-*stereoselectivity, and enantioselectivity (up to 97% ee). Moreover, using two different DES mixtures, the diastereoselection of the process could be tuned, to obtain both the *syn-* and the *anti-*isomer with very high ee values working under different experimental conditions.

DESs were successfully employed as reaction media for continuous production of enantioenriched aldol products, and the excess of cyclohexanone could be recovered and reused. It is worth noting that the reaction can be run and the product isolated without the use of any organic solvent by a proper choice of DES components. The dramatic influence of the reaction media, both on the reaction rate and the stereoselectivity of the process, is consistent with an unprecedented influence of 3D DES architecture on the reactivity of the different species involved in the catalytic cycle, even when using an apparently simple organocatalyst such as L-proline. These observations have important implications in the future design of chiral catalysts, thereby opening the floodgates to new intriguing opportunities for organocatalysis in unconventional reaction media.

## Supporting Information

File 1 Experimental set-up and general procedures for the continuous reactions and in batch reactions; product characterization.

## References

[1] Mahrwald R (2004). Modern Aldol Reactions.

[2] Berkessel A, Gröger H (2005). Asymmetric Organocatalysis.

[3] List B, Lerner R A, Barbas C F (2000). J Am Chem Soc.

[4] Liu X, Lin L, Feng X (2009). Chem Commun.

[5] Orlandi M, Benaglia M, Raimondi L, Celentano G (2013). Eur J Org Chem.

[6] Guizzetti S, Benaglia M, Pignataro L, Puglisi A (2006). Tetrahedron: Asymmetry.

[7] Cho E, Kim T H (2014). Tetrahedron Lett.

[8] Karmakar A, Maji T, Wittmann S, Reiser O (2011). Chem – Eur J.

[9] Opalka S M, Steinbacher J L, Lambiris B A, McQuade D T (2011). J Org Chem.

[10] El-Hamdouni N, Companyó X, Rios R, Moyano A (2010). Chem – Eur J.

[11] Reis O, Eymur S, Reis B, Demir A S (2009). Chem Commun.

[12] Mandal T, Zhao C-G (2008). Angew Chem, Int Ed.

[13] Clarke M L, Fuentes J A (2007). Angew Chem, Int Ed.

[14] Rodriguez B, Bruckmann A, Bolm C (2007). Chem – Eur J.

[15] Clegg W, Harrington R W, North M, Pizzato F, Villuendas P (2010). Tetrahedron: Asymmetry.

[16] Mase N, Nakai Y, Ohara N, Yoda H, Takabe K, Tanaka F, Barbas C F (2006). J Am Chem Soc.

[17] Hayashi Y, Sumiya T, Takahashi J, Gotoh H, Urushima T, Shoji M (2006). Angew Chem, Int Ed.

[18] Guizzetti S, Benaglia M, Raimondi L, Celentano G (2007). Org Lett.

[19] Mlynarski J, Bas S (2014). Chem Soc Rev.

[20] Ilgen F, König B (2009). Green Chem.

[21] Müller C R, Meiners I, Domínguez de María P (2014). RSC Adv.

[22] Martínez R, Berbegal L, Guillena G, Ramón D J (2016). Green Chem.

[23] Massolo E, Palmieri S, Benaglia M, Capriati V, Perna F M (2016). Green Chem.

[24] Kondo K, Yamano T, Takemoto K (1985). Makromol Chem.

[25] Sakthivel K, Notz W, Bui T, Barbas C F (2001). J Am Chem Soc.

[26] Benaglia M, Celentano G, Cozzi F (2001). Adv Synth Catal.

[27] Calderón F, Fernández R, Sánchez F, Fernández-Mayoralas A (2005). Adv Synth Catal.

[28] Smith E L, Abbott A P, Ryder K S (2014). Chem Rev.

[29] Liu P, Hao J-W, Mo L-P, Zhang Z-H (2015). RSC Adv.

[30] Ruß C, König B (2012). Green Chem.

[31] Gu Y, Jérôme F (2013). Chem Soc Rev.

[32] Francisco M, van den Bruinhorst A, Kroon M C (2013). Angew Chem, Int Ed.

[33] Alonso D A, Baeza A, Chinchilla R, Guillena G, Pastor I M, Ramón D J (2016). Eur J Org Chem.

[34] Mallardo V, Rizzi R, Sassone F C, Mansueto R, Perna F M, Salomone A, Capriati V (2014). Chem Commun.

[35] Sassone F C, Perna F M, Salomone M, Florio S, Capriati V (2015). Chem Commun.

[36] García-Álvarez J, Hevia E, Capriati V (2015). Eur J Org Chem.

[37] Cicco L, Sblendorio S, Mansueto R, Perna F M, Salomone M, Florio S, Capriati V (2016). Chem Sci.

[38] Mancuso R, Maner A, Cicco L, Perna F M, Capriati V, Gabriele B (2016). Tetrahedron.

[39] Capua M, Perrone S, Perna F M, Vitale P, Troisi L, Salomone A, Capriati V (2016). Molecules.

[40] García-Álvarez J, Wypych G (2014). Deep Eutectic Solvents and Their Applications as New Green and Biorenewable Reaction Media. Use, Health, and Environment.

[41] Hammond O S, Bowron D T, Edler K J (2016). Green Chem.

[42] Orlandi M, Ceotto M, Benaglia M (2016). Chem Sci.

[43] Martínez-Castañeda A, Rodríguez-Solla H, Concellón C, del Amo V (2012). J Org Chem.

