# Supplementary material for: Towards the development of continuous, organocatalytic, and stereoselective reactions in deep eutectic solvents
Source: Beilstein J Org Chem. 2016 Dec 5;12:2620–6. doi: 10.3762/bjoc.12.258 (PMC5238568; doi:10.3762/bjoc.12.258)
Supplement: File 1 — Experimental set-up and general procedures for the continuous reactions and in batch reactions; product characterization. [file Beilstein_J_Org_Chem-12-2620-s001.pdf]

Supporting Information

for

Towards the development of continuous, organocatalytic, and stereoselective reactions in deep eutectic solvents

Davide Brenna[1], Elisabetta Massolo[1], Alessandra Puglisi[1], Sergio Rossi[1], Giuseppe Celentano[2], Maurizio Benaglia[1]* and Vito Capriati[3]

Address:[1]Dipartimento di Chimica, Università degli Studi di Milano, Via C. Golgi 19, I-20133 Milano, Italy, [2]Dipartimento di Scienze Farmaceutiche, Università degli Studi di Milano, Via Mangiagalli 25, 20133 Milano, Italy and [3]Dipartimento di Farmacia–Scienze del Farmaco, Università di Bari "Aldo Moro", Consorzio C.I.N.M.P.I.S., Via E. Orabona 4, I-70125 Bari, Italy

Email: Maurizio Benaglia - maurizio.benaglia@unimi.it

*Corresponding author

Experimental set-up and general procedures for the continuous reactions and in batch reactions; product characterization

General information	S1
Experimental set up and general procedures for the continuous reactions	S3
General procedure for batch aldol reactions	S6
Products characterization	S7
Mass balance	S13
Recycling experiments	S15

General information

NMR spectra

^{1}H NMR and ^{13}C NMR spectra were recorded with instruments at 300 and 75 MHz respectively (Bruker AMX 300 and Brucker F300). The chemical shifts are reported in ppm (δ), with the solvent reference relative to tetramethylsilane (TMS).

HPLC

For HPLC analyses on chiral stationary phase, to determine enantiomeric excesses, it was used an Agilent Instrument Series 1100. The specific operative conditions for each product are reported from time to time.

TLC

Reactions and chromatographic purifications were monitored by analytical thin-layer chromatography (TLC) using silica gel 60 F254 pre-coated glass plates and visualized using UV light, or stained with phosphomolybdic acid or ninhydrin ethanol solution.

Chromatographic purification

Purification of the products was performed by column chromatography with flash technique (according to the Still method) using as stationary phase silica gel 230–400 mesh (purchased from SIGMA ALDRICH).

Cyclohexanone

Commercially available cyclohexanone was used without any further purification

Aldehydes

Commercially available aldehydes were purified before use, using distillation or crystallization technique

Reactions work-up

The organic phases, if necessary, were dried with Na_2SO_4. The solvents were removed under reduced pressure and then using an high vacuum pump (0.1–0.005 mmHg).

DES

Preparation: The employed deep eutectic solvents (DESs) [DES A choline chloride/urea 1:2; DES D choline chloride/fructose/water 1:1:1; DES E choline chloride/glycerol 1:2)] were prepared by gentle

heating under stirring at 70 °C for 15 min the corresponding individual components until a clear solution was obtained. [DES B and DES C were prepared adding at DES A the stoichiometric amount of water.]

Fluidic device

The device was realized using a normal HPLC pump Gynkotek, High Precision Pump Model 300 and a 100 mL (set-up I) or 10 mL glass cylinder (set-up II and III) according to the scheme reported in Figure S1.

Preparation of the experimental set up and general procedures for the continuous reactions

Procedure 1: (set-up I)

L-Proline (0.35 equiv, 195 mg) was added to the desired DES (1.5 mL) into a small glass cylinder (diameter = 0.5 cm); and the obtained suspension was mixed for 10 min. This cylinder was then placed inside the 100 mL glass cylinder containing a mixture of the desired aldehyde (1 equiv) dissolved in cyclohexanone (20 equiv, 10 mL) (see Figure S1). The pump inlet was introduced in the mixture containing cyclohexanone, and the pump outlet was introduced in the DES-containing glass cylinder. After that, the HPLC pump was turned on with 1 mL/min as flow rate. The reaction was monitored by NMR technique and Chiral HPLC and stopped at the desired time (see article tables). The pump was washed with 3 mL of cyclohexanone, in order to recover the product present in the pump system, the supernatant (cyclohexanone and aldol product) was recovered, and the cyclohexanone was removed by distillation. The crude product was analyzed and eventually purified by column chromatography.

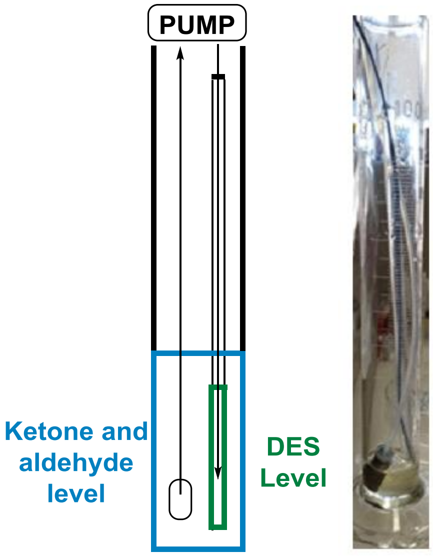

Figure S1

Procedure 2: (set-up II)

L-Proline (0.35 equiv, 195 mg) was added to the desired DES (1.5 mL) into a 10 mL glass cylinder, then a mixture of cyclohexanone (20 equiv, 10 mL) and the selected aldehyde (1 equiv) was slowly added on the top of the DES phase. (Figure S2) The pump inlet was introduced in the cyclohexanone phase and the pump outlet was introduced in the DES phase (see Figure S2). After that, the HPLC pump was turned on with 1 mL/min as flow rate. The reaction was monitored by NMR technique and Chiral HPLC and stopped at the desired time (see article tables). The pump was washed with 3 mL of cyclohexanone, in order to recover the product present in the pump system, the supernatant (cyclohexanone and aldol product) was recovered, and the cyclohexanone was removed by distillation. The crude product was analyzed and eventually purified by column chromatography.

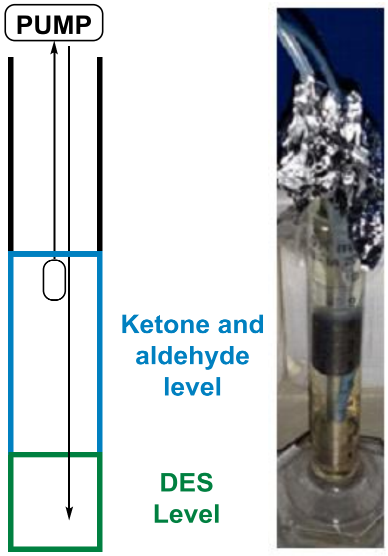

Figure S2

Procedure 3: (set-up III)

L-Proline (0.35 equiv, 195 mg) was added to the desired DES (1.5 mL) into a 10 mL glass cylinder, then N_2 (1 Barr) was bubbled using a tube HPLC connection. Cyclohexanone (20 equiv, 10 mL) and the selected aldehyde (1 equiv) was then slowly added on the top of the DES phase (Figure S3). After that, the HPLC pump was turned on with 1 mL/min as flow rate. The reaction was monitored by NMR technique and chiral HPLC and stopped at the desired time (see article tables). The pump was washed with 3 mL of cyclohexanone, in order to recover the product present in the pump system, the supernatant (cyclohexanone and aldol product) was recovered, and the cyclohexanone was removed by distillation. The crude product was analyzed and eventually purified by column chromatography.

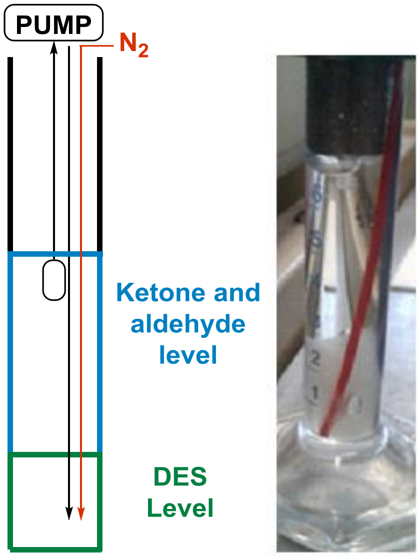

Figure S3

General procedure for batch aldol reactions

In a typical procedure, the desired DES (0.1 mL) and L-proline (0.35 equiv 13 mg) were charged in a 5 mL one round bottomed flask, and stirred for 3 minutes. Cyclohexanone (20 equiv 680 μL) and the aldehyde (1 equiv) were then added in one portion. After 20 hours, 5 mL of water and 5 mL of ethyl acetate were added to the reaction mixture. The two phases were separated and the aqueous phase was extracted with ethyl acetate (2 × 5 mL). The combined organic phases were dried over Na_2SO_4 and the solvent was removed under reduced pressure. The resulting crude mixture was analyzed with NMR and HPLC.

Products characterization

2-[Hydroxy(4-nitrophenyl)methyl]cyclohexan-1-one

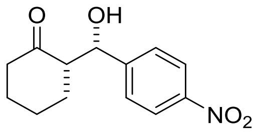

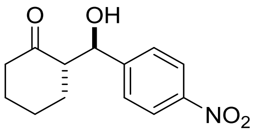

Compound 1 was prepared according to procedures 1, 2 and 3. It was obtained as yellow solid in a mixture of the two diasteroisomers:

Syn: ^{1}H NMR (300 MHz, $CDCl_3$) δ 8.23 (d, *J* = 8.7 Hz, 2H), 7.53 (d, *J* = 8.7 Hz, 2H), 5.50 (s, 1 H),3.19 (br, 1H), 2.68 – 2.29 (m, 3H), 2.19 – 2.07 (m, 1H), 1.87 (m, , 1H), 1.78 – 1.53 (m, 4H).

Anti: ^{1}H NMR (300 MHz, $CDCl_3$) δ 8.23 (d, *J* = 8.7 Hz, 2H), 7.53 (d, *J* = 8.7 Hz, 2H), 4.92 (d, *J* = 8.3 Hz, 1H), 4.11 (s, 1H), 2.68 – 2.29 (m, 3H), 2.19 – 2.07 (m, 1H), 1.87-1.53 (m, *J* , 4H), 1.44-1.32 (m, 1H).

HPLC: The enantiomeric excess of the product was evaluated using OJ-H Chiralcel column, 7:3 Hex:IPA as eluent; flow rate: 0.8 mL/min. $\lambda =$ 210 nm: 11.8 *anti* (major), 12.6 *syn* (minor), 13.8 *anti* (minor), 19.7 *syn* (major).

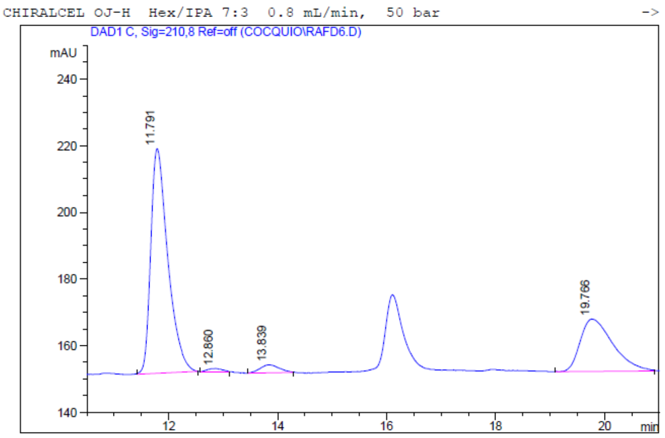

Signal 1: DAD1 C, Sig=210,8 Ref=off

Peak #	RT [min]	Type	Width [min]	Area	Area %	Name
1	11.791	BB	0.321	1462.055	66.769	
2	12.860	MM	0.307	17.064	0.779	
3	13.839	MM	0.414	60.319	2.755	
4	19.766	BB	0.489	650.284	29.697	

2-[Hydroxy(4-chlorophenyl)methyl]cyclohexan-1-one

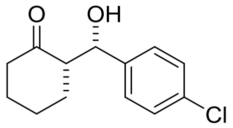

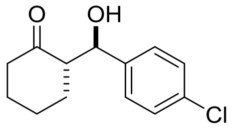

Compound 2 was prepared according to procedures 1,2 and 3: It was obtained as yellow solid in a mixture of the two diasteroisomers:

Syn: ^{1}H NMR (300 MHz, CDCl3) δ 7.34 (d, J = 8.5 Hz, 2H), 7.28 (d, J = 8.5 Hz, 2H), 5.38 (s, 1H), 2.60-2.45 (m, 2H), 2.39-2.09 (s, 2H), 1.98 – 1.42 (m, 3H), 1.31 (d, J = 18.1 Hz, 2H).

Anti: ^{1}H NMR (300 MHz, $CDCl_3$) δ 8.23 (d, *J* = 8.7 Hz, 2H), 7.53 (d, *J* = 8.7 Hz, 2H), 4.92 (d, *J* = 8.3 Hz, 1H), 4.11 (s, 1H), 2.68 – 2.29 (m, 3H), 2.19 – 2.07 (m, 1H), 1.87-1.53 (m, *J* , 4H), 1.44-1.32 (m, 1H).

HPLC: The enantiomeric excess of the product was evaluated using AD chiralcel column, 9:1 Hex:IPA as eluent; flow rate: 0.8 mL/min. $\lambda = 230$ nm: 8.5 *syn* (minor), 10.1 *syn* (major), 13.2 *anti* (major), 15.5 *anti* (minor).

CHIRALCEL AD Hex/IPA 9:1 1 mL/min, 25 bar ->

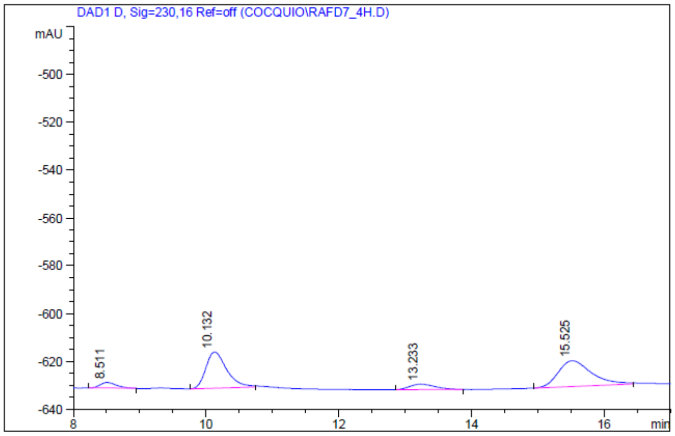

Signal 1: DAD1 D, Sig=230,16 Ref=off

Peak #	RT [min]	Type	Width [min]	Area	Area %	Name
1	8.511	BB	0.260	40.316	4.994	
2	10.132	BB	0.332	332.148	41.143	
3	13.233	BB	0.396	60.865	7.539	
4	15.525	BB	0.508	373.976	46.324	

2-[Hydroxy(4-bromophenyl)methyl]cyclohexan-1-one

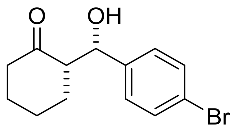

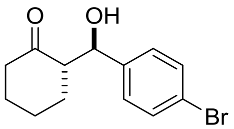

Compound 3 was prepared according to procedures 1,2 and 3: It was obtained as a yellow solid as mixture of the two diasteroisomers.

Syn: ^{1}H NMR (300 MHz, CDCl3) δ 7.45 (d, J = 8.5 Hz, 2H), 7.15 (d, J = 8.5 Hz, 2H), 5.38 (s, 1H), 2.60-2.45 (m, 3H), 2.09 (s, 2H), 1.80 – 1.50 (m, 4H).

Anti: ^{1}H NMR (300 MHz, $CDCl_3$)) δ 7.45 (d, J = 8.5 Hz, 2H), 7.15 (d, J = 8.5 Hz, 2H), 4.72 (d, 1H, J=9 Hz), 2.60-2.45 (m, 3H), 2.09 (s, 2H), 1.80 – 1.50 (m, 4H).

HPLC: The enantiomeric excess of the product was evaluated using AD chiralpak column, 9:1 Hex:IPA as eluent, flow rate: 0.8 mL/min. $\lambda =$ 210 nm: 8.5 *syn* (minor), 10.3 *syn* (major), 13.13 *anti* (minor), 16.0 *anti* (major).

CHIRALPAK AD Hex/iPrOH 9:1 1 mL/min 25 Bar ->

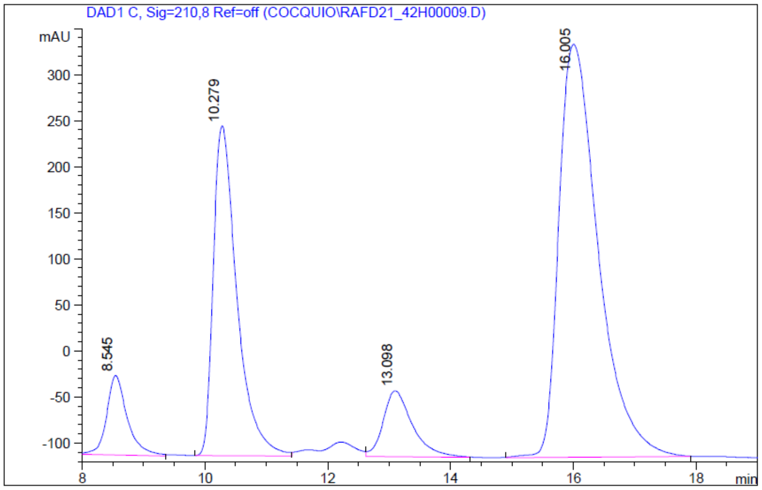

Signal 1: DAD1 C, Sig=210,8 Ref=off

Peak #	RT [min]	Type	Width [min]	Area	Area %	Name
1	8.545	MM	0.391	2026.293	5.966	
2	10.279	BB	0.404	9573.406	28.189	
3	13.098	VB	0.482	2384.760	7.022	
4	16.005	BB	0.676	19976.721	58.822	

2-(Hydroxy(phenyl)methyl)cyclohexan-1-one

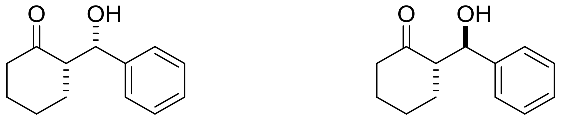

Compound 4 was prepared according to procedures 1,2 and 3: It was obtained as a yellow solid as mixture of the two diasteroisomers.

Syn: ^{1}H NMR (300 MHz, CDCl3) δ 7.39-7.24 (m, 5H), 5.37 (s, 1H), 2.60-2.40 (m, 3H), 2.09–1.98 (m, 1H), 1.88-1.26 (m, 5H).

A*nti*: ^{1}H NMR (300 MHz, CDCl3) δ 7.39-7.24 (m, 5H), 4.78(d, 8.3Hz 1H), 2.60-2.40 (m, 3H), 2.09–1.98 (m, 1H), 1.88-1.26 (m, 5H).

HPLC: The enantiomeric excess of the product was evaluated using OD-H chiralcel column, 95:05 Hex:IPA as eluent, flow rate: 0.5 mL/min. λ = 210 nm: 15.6 *syn* (major), 17.5 *syn* (minor), 21.2 *anti* (major), 31.6 *anti* (minor).

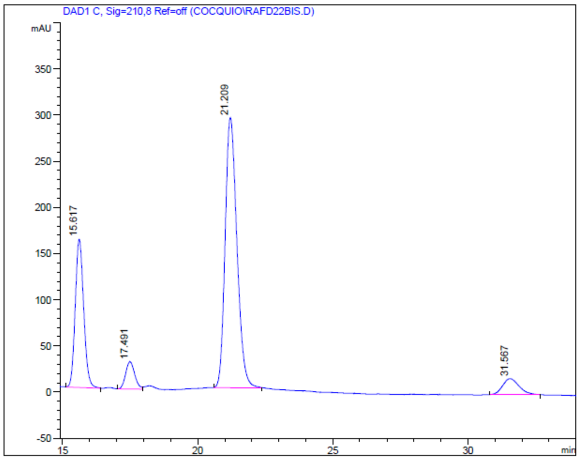

Customized Report: Short

Sorted By Signal
Calib. Data Modified : Thu, 1. Jan. 1970, 01:00:00 am
Multiplier : 1.000000
Dilution : 1.000000
Uncalibrated Peaks : not reported

Signal 1: DAD1 C, Sig=210,8 Ref=off

Peak #	RT [min]	Type	Width [min]	Area	Area %	Name
1	15.617	BB	0.339	3487.790	25.254	
2	17.491	VV	0.336	694.571	5.029	
3	21.209	BB	0.464	8876.351	64.271	
4	31.567	BB	0.515	752.061	5.445	

2-[Hydroxy(2-nitrophenyl)methyl]cyclohexan-1-one

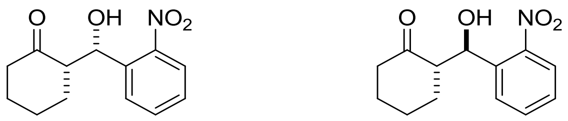

Compound 5 was prepared according to procedures 1, 2 and 3: It was obtained as a yellow solid in a mixture of the two diasteroisomers. Pure *anti* and *syn* diastereoisomers were obtained after chromatographic purification using 7:3 hexane:AcOEt as eluent.

Syn: ^{1}H NMR (300 MHz, CDCl3) δ 8.00 (d, J = 8.2 Hz, 1H), 7.84 (d, J = 8.0 Hz, 1H), 7.64 (t, J = 7.6 Hz, 1H), 7.44 (d, J = 8.3 Hz, 1H), 5.97 (d, J = 2.1 Hz, 1H), 2.88 (dd, J = 12.6, 5.7 Hz, 1H), 2.46 (d, J = 5.3 Hz, 2H), 2.13 (s, 1H), 1.98 – 1.49 (m, 5H).

Anti: ^{1}H NMR (300 MHz, CDCl3) δ 7.80 (dd, J = 21.6, 8.1 Hz, 2H), 7.64 (d, J = 7.5 Hz, 1H), 7.42 (t, J = 7.8 Hz, 1H), 5.44 (d, J = 7.1 Hz, 1H), 2.86 – 2.67 (m, 1H), 2.41 (ddd, J = 31.9, 11.1, 4.6 Hz, 2H), 2.22 – 2.04 (m, 1H), 1.96 – 1.50 (m, 4H), 1.35 – 1.18 (m, 1H).

HPLC: The enantiomeric excess of the product was evaluated using OJ-H chiralcel column, 95:5 Hex:IPA as eluent, flow rate: 0.8 mL/min. ***anti* isomer** λ = 210 nm: 20.3 (minor), 22.1 (major).

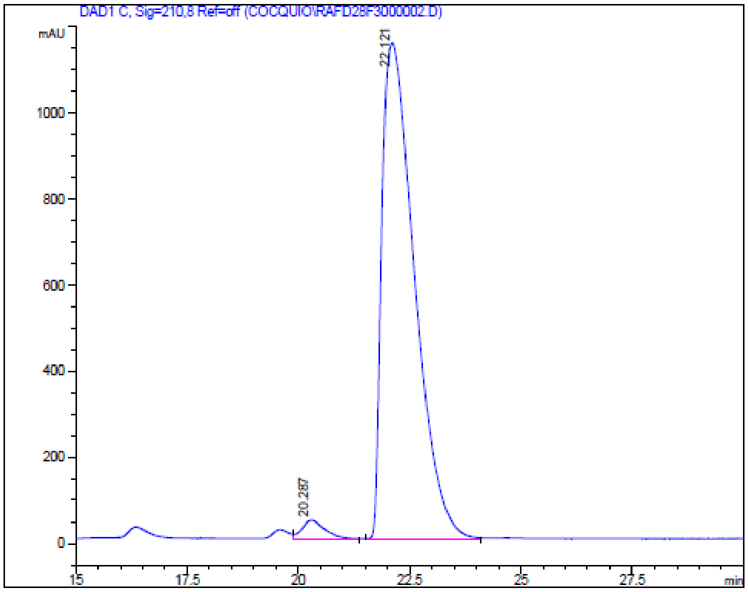

===
Customized Report: Short
===

Sorted By Signal
Calib. Data Modified : Thu, 1. Jan. 1970, 01:00:00 am
Multiplier : 1.000000
Dilution : 1.000000
Uncalibrated Peaks : not reported

Signal 1: DAD1 C, Sig=210,8 Ref=off

Peak #	RT [min]	Type	Width [min]	Area	Area %	Name
1	20.287	VV	0.417	1528.314	2.541	
2	22.121	MM	0.847	58609.262	97.459	

***syn* isomer** λ = 210 nm:: 15.0 (major), 17.0 (minor)

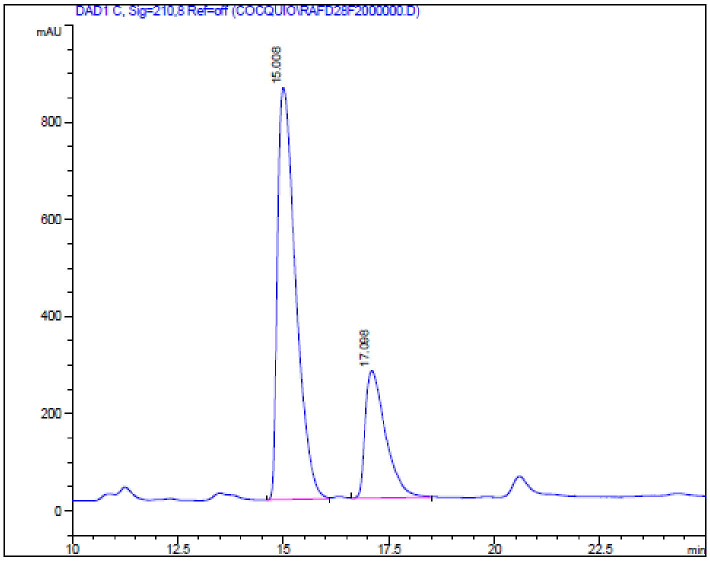

===
Customized Report: Short
===

Sorted By Signal
Calib. Data Modified : Thu, 1. Jan. 1970, 01:00:00 am
Multiplier : 1.000000
Dilution : 1.000000
Uncalibrated Peaks : not reported

Signal 1: DAD1 C, Sig=210,8 Ref=off

Peak #	RT [min]	Type	Width [min]	Area	Area %	Name
1	15.008	BV	0.419	25318.695	74.332	
2	17.098	VB	0.487	8742.714	25.668	

Mass Balance

In Table S1 the conversion, evaluated by ^{1}H NMR on the crude reaction mixture, after cyclohexanone removal, is reported. When the conversion was not confirmed by the weight of the recovered product, such as in the case of aldol **2** (90% conversion, 40% of isolated product), the DES phase was extracted once with 10 mL of ethyl acetate and all the product that was kept in the DES was quantitatively recovered.

Aldol	DES	Conversion (%)	Recovered Product (%)
4-Nitro der. (**1**)	A	99	95
4-Nitro der. (**1**)	B	99	99
4-Cl der. (**2**)	A	90	40
4-Cl der. (**2**)	B	87	56
4-Br der. (**3**)	A	99	95
2-Nitro der. (**5**)	B	38	99

Table S1

The ^{1}H NMR spectra of the crude product after cyclohexanone removal is reported below. No byproducts were observed.

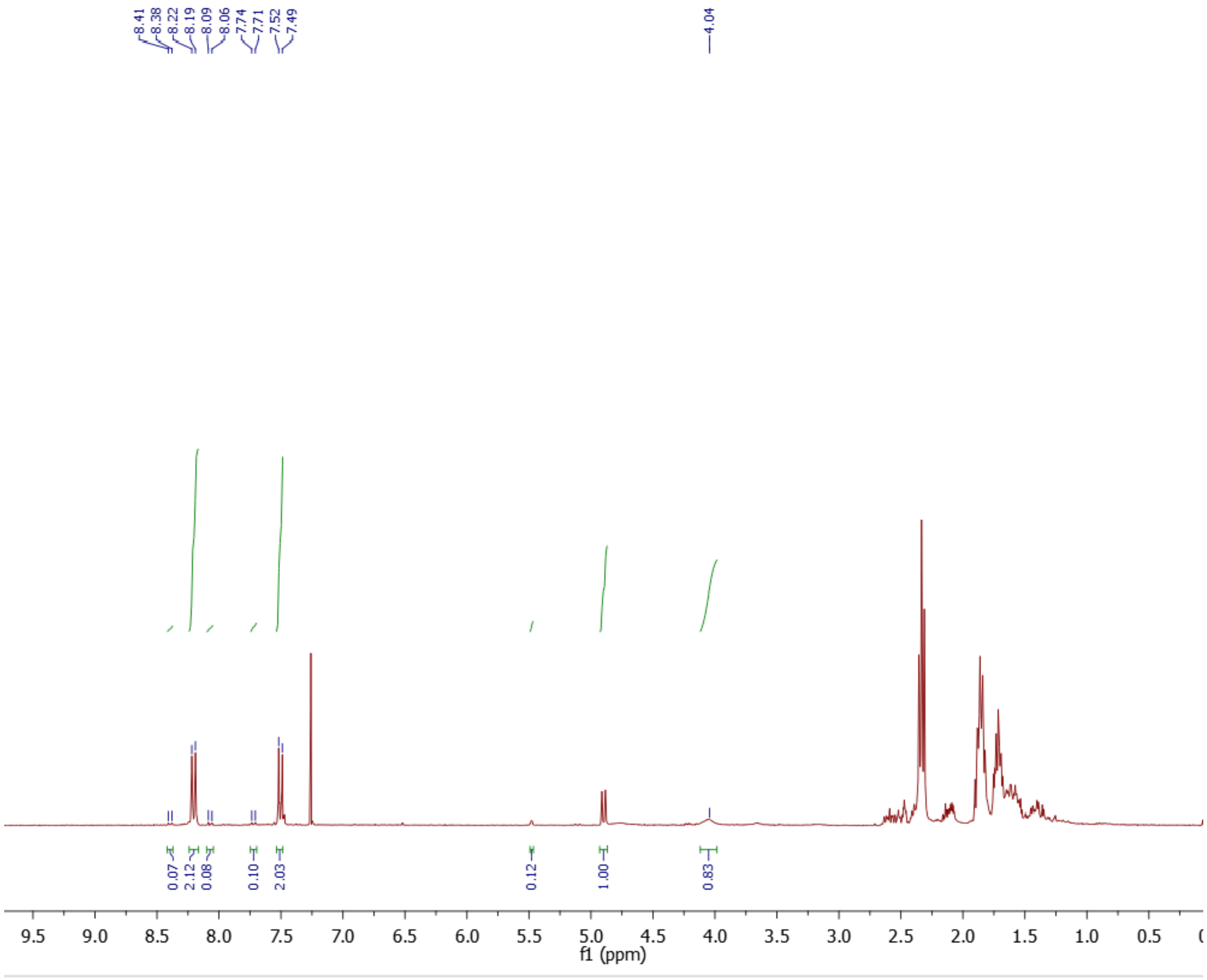

8.41
8.38
8.22
8.19
8.09
8.06
7.74
7.71
7.52
7.49
4.04
0.07
2.12
0.08
0.10
2.03
0.12
1.00
0.83
9.5
9.0
8.5
8.0
7.5
7.0
6.5
6.0
5.5
5.0
4.5
4.0
3.5
3.0
2.5
2.0
1.5
1.0
0.5
f1 (ppm)

Recycling experiments

The DES mixtures A or B (1.5 mL), containing *L*-Proline (0.35 equiv, 195 mg), previously used for 48 hours in the aldol reaction of cyclohexanone with 4-nitrobenzaldehyde, were recycled. At the end of reaction, the pump was washed with 3 mL of cyclohexanone, in order to recover the product present in the pump system, then supernatant (cyclohexanone and aldol product) was separated from the DES phase, containing the catalyst, and analyzed. To the DES phase, new reagents (cyclohexanone and aldehyde) were added and the reaction was started again. While the catalytic systems in DES A showed a lower activity, affording the product in significant low yield, the proline/DESB system afforded comparable results to the first run, both as chemical yield and stereoselectivity.

Reaction in DES mixtures already used in a continuous process

Entry	DES	Time (h)	Conv. (%)[a]	*anti:syn*[a]	ee (%)[b]
1	A	6	5	nd	nd
2	A	22	20	53:47	86-92
3	A	30	23	60:40	85-90
4	A	40	34	57:43	88--92
5	B	2	13	80:20	90-52
6	B	8	30	85:15	93-76
7	B	18	93	70:30	92-68

[a]Conversion and *dr* were evaluated after the cyclohexanone removal on samples taken at indicated reaction times; [b]ee was evaluated using an HPLC with a chiral stationary phase.

Table S2
